# Factors of Power and Equity: Enhancing Our Health System Resilience Research Frameworks: Comment on "Re-evaluating Our Knowledge of Health System Resilience During COVID-19: Lessons From the First Two Years of the Pandemic"

**DOI:** 10.34172/ijhpm.8606

**Published:** 2024-08-12

**Authors:** Stephanie M. Topp

**Affiliations:** College of Public Health, Medical and Veterinary Sciences, James Cook University, Townsville, QLD, Australia.

**Keywords:** Resilience, Health Systems, Research Framework, Governance

## Abstract

The concept of health system resilience has gained prominence in global health discourse, especially in response to the Ebola and COVID-19 pandemics. This commentary responds to Saulnier and colleagues’ 2022 review, which used the Dimensions of Resilience Governance framework to synthesize of COVID-19 related health system resilience research and explore possible conceptual gaps. The review’s findings reveal elements missing from the original framework which underscore the social nature of health systems. This commentary links the review’s empirical findings to nascent theorization of health systems resilience to develop an adapted Framework for Exploratory Research on Health Systems Resilience. A key contribution of the adapted framework is to make explicit the role of actor power and highlight more clearly the distinctions between: (*i*) research focused on identifying the capacities needed to enable adaptation; (*ii*) research focused on the actors whose interests and choices determine which adaptive strategies are used, and (*iii*) research that assess the outcomes of such strategies.

## Introduction

 The concept of health system resilience became a central focus in global health discourse in the wake of the Ebola outbreaks in 2014^1-4^ while the COVID-19 pandemic further intensified attention to, and application of the concept in health and health systems research.^5-9^ A burgeoning literature has since used health systems resilience as an entry point for exploring both the challenges of responding to different types of shock, particularly pandemics and their related impacts, and the capabilities necessary for health systems to adapt effectively under such pressure. Early in the pandemic, for example, Kruk et al^10^ demonstrated how systems with pre-established emergency preparedness measures, such as robust disease surveillance and flexible healthcare workforce strategies, were better equipped to manage the surge in COVID-19 cases. McCollum et al^8^ found that health systems that engaged in active learning from the initial waves of the pandemic applied those lessons to improve response mechanisms in subsequent waves. Yet in these and other instances of pandemic-related resilience research there remained a lack of clarity in relation to both the definition of health systems resilience, and how application of the concept (as distinct from other, normatively defined health system goals such as universal health coverage) might strengthen health system function in different contexts.^11-14^

 In their 2022 article “Re-evaluating Our Knowledge of Health System Resilience During COVID-19: Lessons From the First Two years of the Pandemic” Saulnier et al^15^ conduct a narrative literature review that examines the rapidly expanding body of COVID-19-related health system resilience literature, synthesizing existing knowledge (to date at the time of publication) and identifying gaps. To do so, they use Blanchet and colleagues’ Dimensions of Resilience Governance framework as an organizing heuristic.^16^ This framework suggests that resilience is best understood as an overarching *capacity* comprised of three sub-types – absorptive, adaptive, and transformative capacities. Both the overarching capacity and sub-types are shaped and characterized by four inter-linking capacities: *knowledge* (the capacity to combine and integrate different forms of knowledge); *uncertainties* (the capacity to anticipate and cope with uncertainties and unplanned events); *interdependence* (the capacity to engage effectively with and handle multiple and cross-scale dynamics); and *legitimacy* (the capacity to develop socially and contextually accepted institutions and norms).

 In reviewing the COVID-19 literature, Saulnier et al demonstrate a body of empirical evidence that largely confirms the relevance of the framework’s four interlinking capacities, each understood as contributing to the ability of a health system to absorb, adapt and transform when exposed to shock (such as COVID-19) while still retaining control of its structure and function. But what is most informative and, in this writer’s opinion, field-building about this review, is the analysis of factors or constructs present in the empirical literature yet *missing* from the original framework. These factors notably include: (*i*) explicit consideration of equity and fairness, (*ii*) the influence of values, (*iii*) actor legitimacy, and (*iv*) governance of the private sector.

 The remainder of this commentary will therefore focus on these “missing values” reflecting on their implications for our understanding of health systems resilience in the context of ongoing conceptual debates, and based on this, proposing an adapted framework that builds out from the original.

## The Missing Values of Equity and Fairness

 The first point to discuss is the authors’ observation that, although lacking explicit mention in the Dimensions of Resilience Governance framework, equity is in fact linked to all the dimensions listed. The empirical literature, they observe, raises questions around the comparative value of, and interactions between, the four dimension, given that “the pandemic has shown system weaknesses and existing disparities in accessing and receiving care along social and economic lines.” Some groups have been disproportionately affected by COVID-19 and the indirect effects of related interventions and lock downs. And the review thus flags how resilience capacities, in an of themselves, do not produce universally positive or ethical outcomes.

 Here the review provides empirical support to what others have argued theoretically.^17-19^ That is, that health systems resilience, understood as a *capacity*, is quite distinct from normatively defined health system outcomes such as universal health coverage, or equity of access, or financial protection.^20^ These latter constructs are grounded in ethical principles such as fairness and justice. Health system resilience, on the other hand, is a capacity that contains no guarantee of ethical outcomes.^17,20^ Although not a central focus of their analysis, Saulnier et al do illustrate this point empirically, with examples of desirable and undesirable outcomes arising from (particularly) absorptive and adaptive capacities in action. For example, the rapid expansion of hospital capacities to treat COVID-19 patients which relied on redirecting resources from other parts of the health system, leading to compromised long-term health outcomes for non-COVID-19 patients. This adaptive strategy maintained hospital functionality in many settings, but at a significant ethical and social cost, including overburdening certain segments of the workforce.^15^ A critical framework for understanding health system resilience must therefore differentiate between the *capacity* for resilience, and the ethical implications of its *outcomes *at a system level.

 The implications of these observations for the Dimensions of Resilience Governance framework are significant. The review authors tentatively conclude that equity and fairness may be missing from the framework. However, I would suggest that equity and fairness are not inherently part of the concept of resilience. Health system resilience, as a composite set of capacities, can produce both equity-enhancing and equity-obstructing outcomes. The prevalent belief in some health system research discourses, particularly emanating from high income countries, that resilience always leads to positive (eg, equitable health) outcomes conflates resilience as a capacity with resilience as an outcome. This conflation obscures the necessity to separately evaluate who benefits from resilience responses and who does not, a point that will be elaborated further below. This separation makes it possible to apply the framework to examine mixed and private sector health systems (another “missing value” identified by the author) since it removes any assumption that private sector entities must align with public health goals during emergencies.

 In sum, if we accept resilience as a capacity, the framework‘s agnosticism regarding the positive or negative (equitable or inequitable) impacts of absorptive, adaptive, or transformative responses is appropriate. The focus on different capacities contributing to these adaptations is justified. We must acknowledge that inequity, although undesirable, is a possible and valid outcome of resilient health systems.

## The Missing Influence of Values Actor Legitimacy and the Bigger Issue of “Power”

 The review also identifies additional areas missing from the original framework, specifically the influence of values and actor legitimacy. Strong evidence shows that individual, institutional, and societal values significantly shaped decisions around COVID-19 responses and objectives. This mirrors evidence of the foundational role of values in shaping health systems more broadly.^21^ For instance, the prioritisation of certain services for specific groups during lockdown was both a values-based judgment and a technical decision. The authors note that such decisions were often made under conditions of great uncertainty, frequently without guidelines or ethical frameworks, and without a full understanding of the likely outcomes or consequences. Yet they also highlight that subsystems neglected by pre-pandemic resourcing decisions emerged as weak links in the COVID-19 response, setting the stage for future crises.^15^

 The review of empirical literature also illustrated how perceptions of actor legitimacy influenced the behavior of health providers responsible for implementing health system actions. Individuals and professional groups excluded from decision-making or information flows, such as community health workers or nursing home personnel, often had to adapt to the shock of COVID-19 with comparatively less authority or resources than those with more power. This exacerbated pre-existing challenges, including inequitable conditions and remuneration, leading to resentment, decreased trust, and reduced willingness to cooperate.

 All of these “missing areas” highlight a key element perhaps implicit but never explicit in the Dimensions of Resilience Governance framework: actor power. Adapting the original framework (Figure), power should fill the space around and mediate the interactions *between* the four dimensions of knowledge, uncertainty, inter-dependence, and legitimacy. Recognising relationships of power as the backdrop against which resilience capacities are enacted is important conceptually, because it makes explicit the social nature of health systems, and explains how value-laden norms, practices and relationships come to influence COVID-19 (and other) adaptive strategies and responses. It enhances our understanding of health system resilience as not merely the capacities by which health systems endure and adapt but also the manner and principles according to which these adaptations are executed.^18,22^

**Figure:**
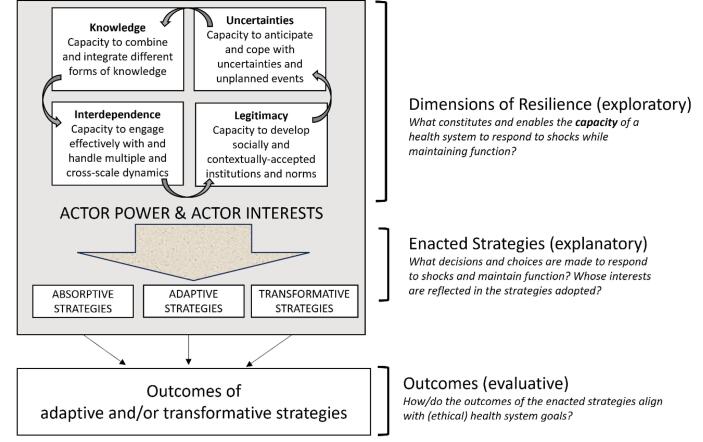
Framework for Exploratory Research on Health Systems Resilience: An Adaptation of the Dimensions of Health Systems Resilience Framework.

 Recognising power (sources, channels and relationships^23,24^) as the backdrop against which resilience capacities are enacted also helps to make sense of the review’s findings viz. equity and the assertion that resilience can produce both positive and negative outcomes. For example, it helps explain how and why certain adaptations benefit some actors, while creating negative effects or outcomes for others.^19^ Although empirical work examining the links between adaptations made by powerful actors and health and service outcomes using a resilience framing is still nascent, Saulnier and colleagues’ review illustrates the potential for such by observing the influence of *values* and *actor legitimacy* in shaping the choice of strategies in the COVID-19 context. The authors observed, for instance, that although the public sector was responsible for running the COVID-19 response in most countries, private sector actors and their different interests played a major role in the ability of many health systems to coordinate a response and maintain routine healthcare.^15^ This provides early empirical support for the idea that actors differentially invoke their power to shape resilience capacities toward certain endpoints.

## Adapting the Original: A Guide for Exploratory Research on Health Systems Resilience

 Recognition of the difference between *capacities* and *strategies *is already present in important work by Gilson et al^25^ who similarly observe that resilience is a process not an end-state and in which mid-level managers, and central-level actors play a critical role. Figure presents an adapted version of Blanchet and colleagues’ framework that aims to make these elements more explicit, centring the role of actor power and interests as a critical backdrop to the ways in which the four dimensions of resilience evolve and interact; and fundamentally influencing the decisions and choice of adaptive strategies made in response to system shocks. As reflected to the right of the figure – this helps make clear three important, but distinct groupings of *research questions* that would add value and provide more clarity in relation to future health systems resilience research. First, in relation to the dimensions of resilience, research that explores: *What constitutes and enables the capacity of a health system to respond to shocks while maintaining function? *Second, in relation to the enacted strategies, research that examines: *what decisions and choices are made in the selection of strategies to respond to the shock, and whose interests are reflected in those strategies? *And third, in relation to the outcomes, research that examines: *how or do the outcomes of enacted resilience strategies align with ethical health system goals*?

## Conclusion

 Saulnier and colleagues’ review identified some gaps and areas of mismatch between findings of empirical research on health systems resilience during the COVID-19 pandemic, and the Dimensions of Resilience Governance framework. Further exploration of those “missing areas” provides insights into the different ways in which health system resilience is invoked as a concept. Drawing out these findings, and linking them to theorization of health systems resilience published since the review, this commentary proposes an adapted Framework for Exploratory Research on Health Systems Resilience that explicitly addresses issues of actor power, and highlights the distinctions between *explanatory* research focused on the different capacities needed to promote resilience; *exploratory* research focused on the decisions and choices that result in adaptive strategies being used; and *evaluative* research that assesses the outcomes of such strategies. The commentary and framework aim to contribute to the ongoing conversation on how research on health system resilience can contribute to larger normative goals of health system strengthening.

## Ethical issues

 Not applicable.

## Competing interests

 Author declares that she has no competing interests.
